# MicroPattern: a web-based tool for microbe set enrichment analysis and disease similarity calculation based on a list of microbes

**DOI:** 10.1038/srep40200

**Published:** 2017-01-10

**Authors:** Wei Ma, Chuanbo Huang, Yuan Zhou, Jianwei Li, Qinghua Cui

**Affiliations:** 1 Department of Biomedical Informatics, School of Basic Medical Sciences, Peking University, 38 Xueyuan Road, Beijing, 100191, China; 2 MOE Key Lab of Cardiovascular Sciences, Peking University, 38 Xueyuan Road, Beijing, 100191, China; 3 Department of Mathematics, Huaqiao University, 269 Huabei Road, Quanzhou, Fujian Province, 362021, China; 4 School of Computer Science and Engineering, Hebei University of Technology, 5340 Xiping Road, Tianjin, 300401, China

## Abstract

The microbiota colonized on human body is renowned as “a forgotten organ” due to its big impacts on human health and disease. Recently, microbiome studies have identified a large number of microbes differentially regulated in a variety of conditions, such as disease and diet. However, methods for discovering biological patterns in the differentially regulated microbes are still limited. For this purpose, here, we developed a web-based tool named MicroPattern to discover biological patterns for a list of microbes. In addition, MicroPattern implemented and integrated an algorithm we previously presented for the calculation of disease similarity based on disease-microbe association data. MicroPattern first grouped microbes into different sets based on the associated diseases and the colonized positions. Then, for a given list of microbes, MicroPattern performed enrichment analysis of the given microbes on all of the microbe sets. Moreover, using MicroPattern, we can also calculate disease similarity based on the shared microbe associations. Finally, we confirmed the accuracy and usefulness of MicroPattern by applying it to the changed microbes under the animal-based diet condition. MicroPattern is freely available at http://www.cuilab.cn/micropattern.

The human body houses a huge number of microorganisms which are mainly composed of bacteria, and these microorganisms inhabit a variety of human organs such as mouth, stomach, gastrointestinal tract, urogenital tract, skin and respiratory^1^. In recent years, with the fast development of microbiome and meta-genome sequencing technology, many studies have identified a number of differentially regulated microorganisms under a variety of conditions and these microbes could play an important role in our health and diseases^2^,^3^,^4^. For example, in the obese individuals, it was found that the number of the H_2_-producing *Prevotellaceae* and the H_2_-utilizing methanogenic archaea *Methanobacteriales* increased. It is known that the interspecies H_2_ transfer between bacterial and archaeal species is an important mechanism for increasing energy uptake by human large intestine in obese individuals^5^. In type 1 diabetes, the butyrate-producing and lactate-utilizing bacteria were reduced^6^. In type 2 diabetes, the number of butyrate-producing bacteria was decreased while the number of sulphate reduction bacteria was increased, and the ratio of *Bacteroidetes* to *Firmicutes* as well as the ratio of *Bacteroides-Prevotella* group to *Clostridium coccoides-Eubacterium rectale* group showed a significantly positive correlation with plasma glucose concentration^7^,^8^. Moreover, it was reported that many environmental factors could affect the components of microbiota. For example, smoking could alter gut microbiota^9^. Different delivery way of infants had different gut microbiota^10^. Different season or diet also had big effects on the components of microbiota^11^,^12^. These findings provided great helps for the understanding of how microbe and human interacted under different condition.

However, currently, computational methods for analyzing the differentially regulated microbes from a microbiome study are limited. Enrichment analysis is one class of important and popular bioinformatics methods in discovering valuable biological patterns and insights from a list of biological items, such as genes, microRNAs, and metabolites etc. For example, DAVID is a web-based tool for enrichment analysis of a list of genes^13^. TAM and MSEA are tools for enrichment analysis of a list of microRNAs and a list of metabolites, respectively^14^,^15^. Currently tools for enrichment analysis of a list of microbes are still not available. We have established a web-based tool named MicroPattern (http://www.cuilab.cn/micropattern) for microbe set enrichment analysis. In addition, MicroPattern also implemented an algorithm we presented previously for the calculation of microbe-based disease similarity^16^.

## Results

### Microbe sets

**Figure 1:**
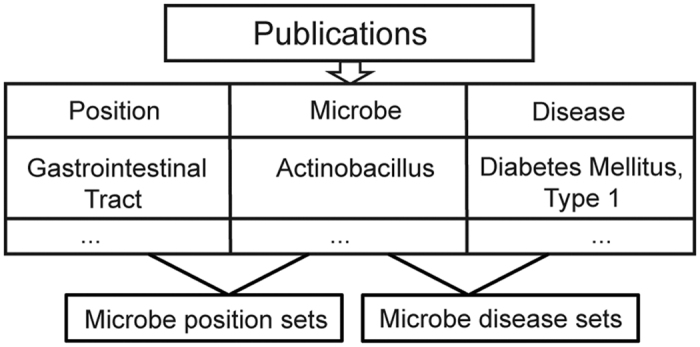
Catalog of microbe set. We grouped microbes that associated with the same disease or colonized on the same body position into the same microbe set. Different microbe sets could overlap with each other.

**Figure 2:**
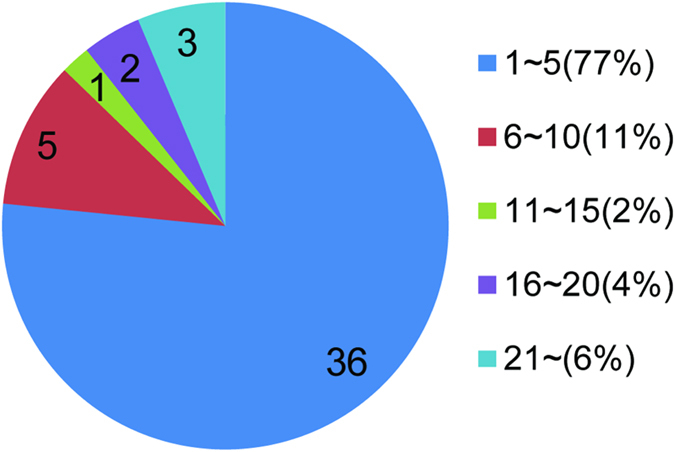
Size distribution of microbe sets. The pie chart indicating the proportion of microbe sets of each size.

In total, 47 microbe sets were collected including 37 disease sets (where microbes in the same set is associated with the same disease) and 10 position sets (where microbes in the same set is colonized on the same body position). In this work, we just keep microbes that in genus or species rank. Thus, two disease sets were abandoned due to lack of such specified microbe association. Flowchart for microbe sets integration was showed in Fig. 1. Among these sets, the size of 36 sets was in the range of 1~5(77%), 5 sets in the range of 6~10(11%), 1 set in the range of 11~15(2%), 2 sets in the range of 16~20(4%) and 3sets in the range of 21~209(6%), see also Fig. 2. All sets can be downloaded from our web server.

### Analysis procedure of MicroPattern

**Figure 3:**
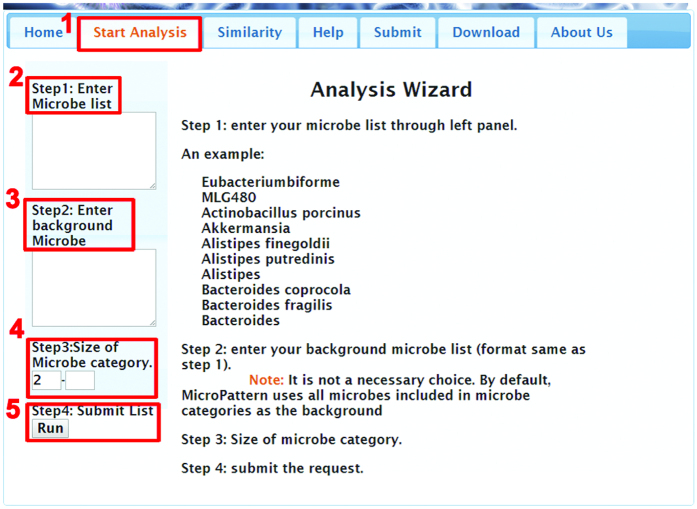
Stepwise guideline for performing the microbe set enrichment analysis.

The procedure for enrichment analysis is illustrated in Fig. 3. MicroPattern works in four steps. In Step 1, a list of interested microbes needs to be inputted. Step 2 is an optional step. The list of microbes inputted in Step 2 will be treated as the background. If a background list is not provided, all microbes in all sets will be used as the background list. In Step 3, the users would choose what sets should be used for analysis according to the size of sets. By default, only the microbe set that includes at least two microbes will be considered. In Step 4, the user can click button “Run” and the result page will be automatically generated after all calculations have been done. In the result page, the microbe set, number of match microbes to this set, percent of match microbes, fold of overrepresentation, Bonferroni value and FDR value are shown. When mouse moves over the name of the microbe set, the matched microbes and non-matched microbes in this set will be listed in a pop-up box. The user can also double click the set name to download the data. Click the button “Bar plot of result” can plot a bar plot.

**Figure 4:**
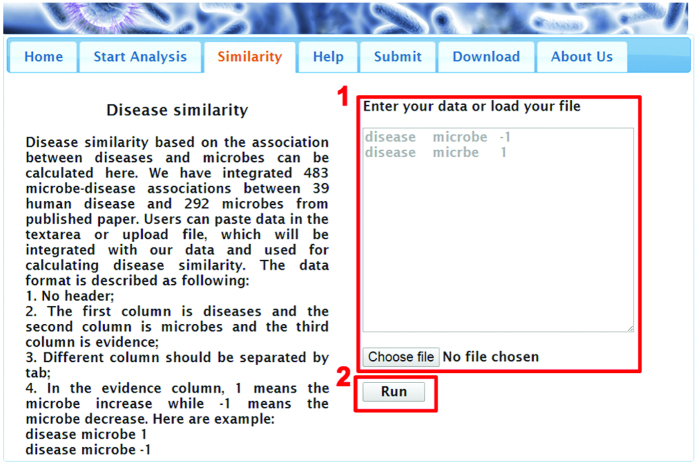
Stepwise guideline for running the disease similarity calculating procedure.

For disease similarity calculation, two steps are need. As shown in Fig. 4, in Step 1, the list of microbe-disease association pairs need to be entered or uploaded. In Step 2, click button “Run” and the result will be shown in a new page. In the result page, the first column and the second column are two diseases and the third column is similarity between them.

Detailed tutorial about how to use MicroPattern are shown on the “Help” page of our web server.

### Diet altering the human gut microbiome, which is associated with disease

We applied MicroPattern to 51 changed microbes (Table 1) from a study screening the changed microbes in human gut after animal-based diet^17^. In this study, 10 American volunteers were involved including 6 male and 4 female. These volunteers were treated with plant-based diet and animal-based diet. Changed microbes were then identified by comparing animal-based diet versus normal diet. For the purpose of investigating the meaningful patterns of these changed microbes, we identified the enriched microbe sets for the changed microbes. As a result, liver cirrhosis was significantly enriched (Table 2; FDR = 2.20 × 10^−6^). This prediction was supported by another study. In this study, high-fat, high-cholesterol diet, which is also common in animal diet, could induce non-alcoholic steatohepatitis and progressing to liver cirrhosis^18^.

## Discussion

With the rapid development of high-throughput biological techniques, more and more studies were focus on microbiome. It was important to identify the relationships between microbe and disease. MicroPattern is tool for predicting associated diseases of changed microbes and calculating disease similarity based on their shared microbe associations. Thus, MicroPattern could figure out how disease and microbe interacted. Moreover, with the accumulation of study focus on human microbiome, more associations between microbe and disease will be curated and MicroPattern will be improved greatly.

## Materials and Methods

### Collection of microbe sets

We searched the microbiome-related articles from Pubmed with the keyword “human microbiome” and manually curated the microbe-disease associations from the literature. In total, we have curated 483 microbe-disease associations from 61 publications. The microbe-disease association was defined as the microbe significantly increase or decrease under disease condition, as judged by the authors of original publications. To be precise and consistent, only the microbes of species and genus ranks were retained. Uncertain associations, if reported, were also omitted. The microbe-disease association dataset includes a total of 39 human diseases and 292 microbes. Here one microbe set is defined as a group of microbes that have the same meaningful association. For example, the microbes associated with one disease will be grouped into a microbe set. We used the union set of associated microbes from different studies for each disease, because current microbiome data are too variable to obtain one consensus microbe set across different studies^19^,^20^,^21^. In addition to the microbe-disease dataset, we also annotated the information for the body positions where the microbes colonized. So current microbe sets were collected according to two rules, the microbe associated disease and the microbe colonized positions. In total, we collected 47 microbe sets including 37 disease-microbe sets and 10 position-microbe sets.

### Enrichment analysis

We used the hypergeometric test^22^ to determine the significant overrepresentation of the microbe sets among a list of microbes of interest. Assuming that *N* represents the number of microbes included in all microbe sets, *n* represents the number of microbes included in the tested microbe set, *M* represents the number of microbes included in the interested microbe list and *m* represents the number of microbes that matched the tested microbe set. The statistical significance of this microbe set overrepresentation among the interest microbes are represented by the following formula:


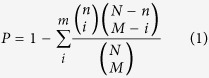


Finally, the P values for all microbe sets are adjusted by Bonferroni and Benjamini-Hochberg FDR corrections.

### Disease similarity calculation

We adapted the equation for the calculation of symptoms-based disease similarity to calculate the microbe-based disease similarity^23^. For every disease *i* (39 in total) and every microbe *j* (292 in total), we described the *w*_*ij*_ as the quantitative strength of relationship between them:


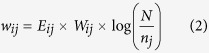


*E*_*ij*_ (*E*_*ij*_ ∈ [−1, 1]) represents the changing direction of microbe *j* in disease *i. E*_*ij*_ equals to 1 when microbe *j* is increased in disease *i*, while *E*_*ij*_ equals to −1 when microbe *j* is decreased in disease *i. W*_*ij*_ represents the number of associations of disease *i* and microbe *j. N* (here is 39) is the number of all disease and *n*_*j*_ is the number of diseases associated with microbe *j*. Thus, for every disease *i*, it has a vector *d*_*i*_ of length *M (M* is the number total microbes, here is 292).

Then we took the cosine similarity value between two vectors *d*_*i*_ and *d*_*j*_ as similarity between disease *i* and disease *j* as


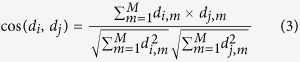


## Additional Information

**How to cite this article:** Ma, W. *et al*. MicroPattern: a web-based tool for microbe set enrichment analysis and disease similarity calculation based on a list of microbes. *Sci. Rep.* **7**, 40200; doi: 10.1038/srep40200 (2017).

**Publisher's note:** Springer Nature remains neutral with regard to jurisdictional claims in published maps and institutional affiliations.

## Figures and Tables

**Table 1:** Significant changed microbes under the animal-based diet condition.

Taxonomic rank	Microbes
Species rank	*Eubacterium biforme,* Microbe MLG480*, *Actinobacillus porcinus, Alistipes finegoldii, Alistipes putredinis, Bacteroides coprocola, Bacteroides fragilis, Bacteroides salyersiae, Bifidobacterium adolescentis, Bifidobacterium gallicum, Bifidobacterium longum, Bilophila wadsworthia, Blautia producta, Clostridium bolteae, Clostridium orbiscindens, Collinsella aerofaciens, Dialister invisus, Faecalibacterium prausnitzii, Megasphaera elsdenii, Mitsuokella multacida, Parabacteroides johnsonii, Prevotella copri, Raoultella, Roseburia Eubacteriumrectale, Roseburia faecis, Ruminococcus bromii, Ruminococcus callidus, Ruminococcus flavefaciens, Ruminococcus gnavus,*
Genus rank	*Alistipes, Akkermansia, Bacteroides, Bifidobacterium, Blautia, Catenibacterium, Clostridium, Coprococcus, Dialister, Escherichia, Eubacterium, Faecalibacterium, Lachnobacterium, Lachnospira, Odoribacter, Oscillospira, Parabacteroides, Phascolarctobacterium, Roseburia, Prevotella, Ruminococcus, Sutterella*

^*^This microbe has no formal species name.

**Table 2:** MicroPattern analysis result for changed microbes under the animal-based diet condition.

Microbe sets	P value	FDR
Disease		
Liver cirrhosis	1.38 × 10^−7^	2.20 × 10^−6^
Clostridium difficile	0.0132	0.079
Irritable bowel syndrome	0.0197	0.079
Arthritis, rheumatoid	0.0367	0.1173
Position		
Gastrointestinal tract	0.0161	0.079
